# TOPSE for babies in Norwegian: examining the reliability of a tool to measure parenting self-efficacy

**DOI:** 10.1017/S1463423625000295

**Published:** 2025-04-24

**Authors:** Marit Burkeland-Lie, Mari Hysing, Anders Dovran, Sally Kendall, Jens Christoffer Skogen

**Affiliations:** 1 PhD candidate, Norwegian Institute of Public Health and University of Bergen, Bergen, Norway; 2 Professor and Clinical Psychologist, Department of Psychosocial Science, University of Bergen, Bergen, Norway; 3 Researcher and Clinical Psychologist, Stine Sofies Foundation, Grimstad, Norway; 4 Professor, University of Kent, Canterbury, United Kingdom; 5 Senior Researcher and Clinical Psychologist, Norwegian Institute of Public Health, Bergen, Norway; 6 Center for Alcohol and Drug Research (KORFOR), Stavanger University Hospital, Stavanger, Norway; 7 Centre for Evaluation of Public Health Measures, Norwegian Institute of Public Health, Oslo, Norway

**Keywords:** Children, infants, parenting, parenting self-efficacy, parents

## Abstract

**Background:**

Parents’ confidence in their parenting abilities, or parenting self-efficacy (PSE), is an important factor for parenting practices. The Tool to measure Parenting Self-Efficacy (TOPSE) is a questionnaire created to evaluate parenting programmes by measuring PSE. Originally, it was designed for parents with children between the ages of 0–6 years. A modified version specifically for parents of infants aged 0-6 months (TOPSE for babies) is currently being piloted. In this study, we translated TOPSE for babies and investigated the reliability of the Norwegian version.

**Aim:**

To investigate the reliability of the Norwegian version of TOPSE for babies.

**Methods:**

The study included 123 parents of children aged 0–18 months who completed a digital version of the TOPSE questionnaire. Professional translators performed the translation from English to Norwegian and a back translation in collaboration with the author group. Mean and standard deviation were calculated for each of the questionnaire’s six domains, and a reliability analysis was conducted using a Bayesian framework for the total sample (parents of children aged 0–18 months) and specifically for the parents of the youngest group of children (0–6 months).

**Findings:**

The Norwegian version of TOPSE for babies is a reliable tool for measuring parenting self-efficacy. However, some variations exist across the children’s age groups and domains. The overall Bayesian alpha coefficient for the suggested domains ranged from 0.54 to 0.83 for the entire sample and from 0.63 to 0.86 for parents with children aged 0–6 months. For two of the domains, one item in each proved to largely determine the low alpha coefficients, and removing them improved the reliability, especially for parents with children aged 0–6 months.

## Introduction

Transitioning into parenthood can elicit strong feelings such as satisfaction, love and responsibility, but also loss of confidence, anxiousness, and a sense of being overwhelmed. Several developmental changes occur in early childhood, and parents can experience many challenging situations. In examining parents’ experiences during the first year after birth, Nyström and Ohrling (2004) found that despite variations between mothers and fathers, they both experienced it as ‘living in a new and overwhelming world’. The review also emphasizes the importance of interventions to minimize strain by empowering parents in their new role. *Parenting self-efficacy* (PSE) is a common target for such interventions. It is defined by Ardelt and Eccles (2001:945) as ‘…parents’ beliefs in his or her ability to influence the child and his or her environment to foster the child’s development and success’.

A review of PSE in parent and child adjustment found that PSE has been positively related to parental monitoring and responsiveness, parenting competence, and satisfaction (Jones and Prinz, 2005). The review also included findings suggesting that PSE is related to child adjustment and that parents with high PSE had more confidence in exercising effective parenting in challenging situations. In a more recent review of factors associated with PSE, Fang et al. (2021) found evidence of an association between PSE and parenting stress, depression, and perceived social support. The significance attributed to PSE has led to the development of interventions targeting PSE to improve the child-rearing environment (Wittkowski et al., 2017). One tool developed to evaluate such interventions is the Tool to measure Parenting Self-Efficacy (TOPSE) (Kendall and Bloomfield, 2005). Given the developmental changes during early childhood, it is imperative to consider potential fluctuations in parents’ PSE levels when evaluating PSE measures.

## Assessing parenting self-efficacy: TOPSE

PSE is usually assessed through self-reported measures, which is suitable considering it reflects parents’ perception of and belief in their parenting abilities (Wittkowski et al., 2017). One such measure is the TOPSE (Kendall and Bloomfield, 2005). Sally Kendall and Linda Bloomfield created it to assess PSE in parents of children aged 0–6 years. The development of TOPSE was based on focus group interviews with parents and healthcare professionals, and the instrument’s psychometric properties were initially investigated in a small-scale study (N=63) (Kendall and Bloomfield, 2005). The study’s participants were parents of children up to the age of six, where the majority were mothers.

Initially, TOPSE comprised 82 items across nine domains: Affection/emotion, Play, Empathy/understanding, Routines/goals, Control, Boundaries, Pressure, Acceptance, and Learning/knowledge. After further development, the questionnaire items were reduced to 48, and the domains were reduced to eight: Emotion and affection, Play and enjoyment, Empathy and understanding, Control, Discipline and boundaries, Pressures, Self-Acceptance, and Learning and knowledge (Bloomfield and Kendall, 2012).

In addition to TOPSE, there are 33 available PSE measures. In a review examining the psychometric properties of such measures, the original TOPSE version with nine domains was included (Wittkowski et al., 2017). The review used the quality rating tool by Terwee et al. (2007) and four criteria from Bot et al.’s (2004) ‘clinimetric’ checklist to assess the psychometric quality of the development and validation work carried out on each measure. The mean score was 12.67, with the highest score being 28 and the lowest being one. TOPSE obtained a total score of 15 out of 36, and received the maximum score of 3 (indicating that the measure has undergone rigorous psychometric evaluations) on Content validity, Reproducibility (agreement), Interpretability and Ease of scoring (Wittkowski et al., 2017). The information found for Time to administer was considered below a specific threshold (receiving a score of 2), and for Construct validity the information found was considered lacking or doubtful (receiving a score of 1). No information was found for the remaining psychometric properties.

For the original validation study, the internal reliability coefficients ranged from 0.80 to 0.89 for the nine subscales, with an overall scale reliability of 0.94 (Kendall and Bloomfield, 2005). In a later study (N=356) including parents, mainly mothers, of children aged six months to 10 years, the alpha coefficient of the nine subscales ranged from 0.65 to 0.89, and the overall scale reliability was 0.89 (Bloomfield and Kendall, 2007). In the study (N=58) using the version with eight subscales and 48 items, the alpha of the domains ranged from 0.78 to 0.90, with an overall scale reliability of 0.91 (Bloomfield and Kendall, 2012).

The version of TOPSE with eight domains seems to be a valid and reliable tool in several languages. In a validation study (N=180) from Bangladesh, which included mothers of children aged 0-6 years old, TOPSE had an acceptable internal consistency (Ferdowshi, Imran and Trishna, 2021). The overall coefficient alpha was 0.89, ranging from 0.81 to 0.91 across the eight domains. A Serbian validation study (N=970) included parents of one or more children between 0–6 years and expecting parents. This study’s coefficient alpha for the domains ranged from 0.62 to 0.86 (Sokolovic, Grujic and Pajic, 2022). The mean age was 34, and the study included 132 fathers.

## Further development and validation of TOPSE

Parents’ PSE levels may change during early childhood development, due to the child’s rapid development and the parents’ changing demands of parenting. Still, many of the available PSE measures are designed for parents of children in a wide age range (Wittkowski et al., 2017). 11 out of 33 are for parents of infants (preterm to 13 months), with eight of them having an age range of 0–5 months. The original version of TOPSE was created for parents of children between 0–6 years (Kendall and Bloomfield, 2005). In later studies (Kendall and Bloomfield, 2007; Bloomfield and Kendall, 2012), older children up to 10 years were included.

Although TOPSE has a wide age range, few validation studies have systematically examined variations in parents’ PSE levels across different age groups of their children. The creators of TOPSE have been contacted by professionals working with parents of infants and asked them to modify TOPSE to enhance its applicability to this group of parents (Kendall, 2023). Therefore, a new version of the tool was developed for parents of infants aged 0-6 months: TOPSE for babies. It excludes the two domains, Control and Discipline and boundaries, which include statements less fitting to parents of infants, such as Item 2 in Control, ‘My child will respond to the boundaries I put in place’, or Item 3 in Discipline and boundaries, ‘I am able to reason with my child’. The remaining six of the eight domains from the 2012 version were retained.

To our knowledge, the first study to use TOPSE for babies is an Italian study, which explored variations in PSE levels across different ages within the first year of a child’s life. In this study, 265 parents of children between 0–12 months were included, of which 131 were fathers (Roncaglia et al., 2023). The participants completed the questionnaire at two weeks, six months, and 12 months after birth. At 2 weeks, the questionnaire excluded the two domains Control and Discipline and boundaries. The coefficient alpha ranged from 0.60 to 0.89 and showed good reliability for five domains, except for Emotion, Self-acceptance, and Learning.

The present study aims to investigate the psychometric properties of the Tool to measure Parenting Self-Efficacy for babies in Norway (TOPSE), as well as the differences across age groups. The inclusion criteria for our study were, therefore, being parents of infants between 0–18 months. To examine possible differences for a wide age span (0–18 months) and 0–6 months specifically, we chose to include parents of children from 7–18 months as well as parents of children between 0-6 months, which is the age group TOPSE for babies was developed for.

## Methods

### Procedure and recruitment

Data collection was conducted between August 2023 and February 2024 using an electronic questionnaire. The data collection was done through TSD (Øvrelid, Bygstad and Thomassen, 2021; University of Oslo, 2024), using the integrated questionnaire solution for collecting data (‘Nettskjema’). All participants were anonymous and gave informed consent in the electronic questionnaire. We used convenience sampling and recruited parents through healthcare centres, kindergartens, and social media. We were aided by the section for public nurses in the Norwegian Nurses Association in reaching out to healthcare centres across the country, and the non-profit foundation Stine Sofies Stiftelse helped us contact several kindergartens. We created an information sheet with a QR-code linking to the questionnaire that the healthcare centres and kindergartens could use to recruit parents. Some also shared the link for the questionnaire on selected social media platforms, such as Stine Sofies Stiftelse’s and some healthcare centres’ platforms. The study also has a project-site on the Norwegian Institute of Public health’s website, where the questionnaire could be accessed through a link and QR-code. We did not ask the participants where they accessed the questionnaire and, therefore, do not know where each participant was recruited.

### Background variables

Our participants (N=123) reported if they were a mother or a father. They could also give information about their age based on the following options: ‘Under 25’, ‘Between 25–30’, and ‘Over 30’. For the children’s age, the options were ‘0–6 months’, ‘7–12 months’, and ‘13–18 months’. We also asked if the participants had caregiver duties for more than one child under the age of 18, with the options being ‘yes’ or ‘no’.

### TOPSE for babies

Within each of the six domains, there are six self-efficacy statements. The statements are identical to the ones in the 2012 version, except for the word ‘child’ being replaced with ‘baby’. Parents are asked to state how much they agree with each statement using a scale from 0–10, where 0 is ‘completely disagree’, 5 is ‘moderately agree’, and 10 is ‘completely agree’ (Sally Kendall, personal communication, February 2023; Kendall, 2023).

### Norwegian translation of TOPSE for babies

In February 2023, we contacted Professor Sally Kendall and requested permission to translate and use TOPSE for babies. Professional translators performed the translation from English to Norwegian and a back translation. A comparison and discussion of discrepancies between the original and back-translated version were conducted by part of the author team: Author 1 (PhD Candidate, Norwegian Institute of Public Health), Author 2 (Professor and Clinical Psychologist, University of Bergen), and Author 5 (Senior Researcher and Clinical Psychologist, Norwegian Institute of Public Health). We communicated with the translators about sentence structures, phrasing, and other linguistic aspects to ensure the highest level of correspondence between the original English version and the Norwegian translation. As described in The World Health Organization’s (2010) protocol for translations, we arrived at a final translation that accurately preserves the intended meaning of the original text.

There is one statement in particular that translated poorly to Norwegian. ‘I am sure my baby can come to me’ translates poorly because ‘come to me’ in Norwegian carries a stronger physical connotation than the English phrase, as it emphasizes the physical act of moving towards someone. However, we included the original translated statement to solve this issue and created another version with different wording. Additionally, there were some words or phrases that changed in the back-translation, such as ‘emotions’, ‘nice’ and ‘assert myself’, which became ‘emotion’, ‘good’ and ‘stand up for myself’. These changes have a limited significance as they would translate similarly to Norwegian.

#### Ethics

The study was presented to the Regional Committees for Medical and Health Research Ethics (REK, #596277). It was considered outside their remit as it was not regarded as health-related research. The data collection and storage have been subject to a Data Protection Impact Assessment (DPIA) by the General Data Protection Regulation (GDPR). We obtained consent from all participating parents through the digital questionnaire. The study adheres to ethical standards of relevant national and institutional guidelines and with the Helsinki Declaration of 1975.

### Statistical analysis

The data were analysed using R version 4.3.0 (R Core Team, 2022). The analyses were conducted for the entire dataset (all parents with children aged 0–18 months) and the subgroup of parents of the youngest children (aged 0–6 months). We calculated each domain’s mean and standard deviation across the six variables constituting a domain, using the rowSums function which omits missing values independently for each row or column.

We analysed each domain’s reliability, including Bayes’ alpha estimates and the corresponding 95% credible intervals. We calculated the probability that the true reliability coefficient exceeds a certain threshold (0.70) to assess the likelihood of achieving a desired level of reliability. Alpha coefficients above 0.70 are commonly considered satisfactory (George and Mallory, 2003). We used the ‘bayesrel’-package (Pfadt, van den Bergh and Goosen, 2023) available in R for this analysis. We opted for a Bayesian framework as it offers a more robust framework for quantifying uncertainty compared to the more traditional approach of relying on Cronbach’s alpha (Pfadt et al., 2023). Bayesian methodology allows for a more nuanced reliability interpretation by quantifying uncertainty and calculating credible intervals. Given our study’s small sample size, a Bayesian analysis is particularly advantageous as it provides more reliable estimates of reliability coefficients in such contexts. Using this framework, we aim to provide a more complete understanding of the reliability of the Norwegian version of TOPSE for babies. Tables were produced using the ‘gtsummary’-package (Sjoberg et al., 2021) and the ‘flextable’-package (Gohel and Skintzos, 2023).

## Results

The study had 123 parents participating, most of whom were mothers (N=113) and over the age of 30 (N=81). Among the participants, 45 (37%) were parents of a child aged 0–6 months, and 82 (66.7%) reported that they had caregiver duties for more than one child under the age of 18 (see Table 1). There were no differences across domains for having caregiver duties for more than one child compared to only one child (all p-values >0.05). The response time in our sample was a median of 6.6 minutes (interquartile range of 4.7 to 10.7).

**Table 1.** Overview of participant’s characteristics (N=123)

Variables	n(%)
Parenting role	
Father	10 (8.1%)
Mother	113 (92%)
More than one child	
Yes	82 (66.7%)
No	41 (33.3%)
Parent age group	
Between 18–30	42 (34%)
Over 30	81 (66%)
Child gender	
Boy	72 (59%)
Girl	51 (41%)
Child age group	
0–6 months	45 (37%)
7–12 months	27 (22%)
13–18 months	51 (41%)

Table 2 presents each domain’s mean scores and reliability analysis results across the total sample. The coefficient alpha ranged from 0.54 to 0.83. Play and enjoyment, Empathy and understanding, Self-acceptance and Pressures all exceeded the desired alpha coefficient (>0.70), while Emotion and Affection and Learning and knowledge did not meet this criterion. One item in each of these domains largely determined the low reliability (see Table 3). When removing Item 6 in Emotion and affection, ‘I find it hard to cuddle my baby’, the alpha increased to 0.59, and the probability increased very slightly to 1.71%. Upon removing Item 6 in Learning and knowledge, ‘Knowing that other people have similar difficulties with their babies makes it easier for me’, the alpha exhibited an increase to 0.75 and a probability of 88.07%.

**Table 2.** Mean and reliability across domains for all parents

Domain	Mean (SD)	Estimate (Bayes)	Lower CI (Bayes)	Upper CI (Bayes)	Prob. >0.7
Emotion and affection	56.0 (4.3)	0.54	0.45	0.64	0
Play and enjoyment	53.7 (5.8)	0.83	0.79	0.87	100.00
Empathy and understanding	53.1 (5.8)	0.80	0.75	0.85	99.89
Pressures	17.3 (11.0)	0.75	0.69	0.82	93.82
Self-acceptance	51.1 (8.1)	0.81	0.77	0.86	100.00
Learning and knowledge	53.5 (6.0)	0.66	0.56	0.75	24.14

**Table 3.** Mean and reliability for domains ‘Emotion and affection’ and ‘Learning and knowledge’ after item removal. All parents

Domain	Mean (SD)	Estimate (Bayes)	Lower CI (Bayes)	Upper CI (Bayes)	Prob. >0.7
Emotion and affection Item 6 removed	47.0 (2.6)	0.59	0.49	0.70	1.72
Learning and knowledge Item 6 removed	44.7 (5.3)	0.75	0.67	0.82	88.07

As shown in Table 4, the alpha for the parents of children aged 0-6 months ranged from 0.63 to 0.86, where Play and enjoyment, Empathy and understanding, and Self-acceptance showed an alpha coefficient exceeding the desired level. The Pressures domain had an alpha coefficient of 0.69, almost reaching the desired level. Emotion and affection and Learning and knowledge did not meet the criterion of an alpha coefficient above 0.70. Emotion and affection had an alpha of 0.63 and a probability of 17.86% for being above 0.70. When removing Item 6 from the domain (see Table 5), the alpha increased to 0.71, with a probability of 62.84%. The Learning and knowledge domain had an alpha of 0.67, with a probability of 39.61%. Removing Item 6 in this domain increased the alpha to 0.75 and the probability to 82.77%. We could not identify any specific items negatively impacting the alpha estimates for the Pressures domain.

**Table 4.** Mean and reliability for parents of children aged 0–6 months

Domain	Mean (SD)	Estimate (Bayes)	Lower CI (Bayes)	Upper CI (Bayes)	Prob. >0.7
Emotion and affection	55.6 (4.7)	0.63	0.47	0.77	17.86
Play and enjoyment	53.5 (6.4)	0.86	0.80	0.91	100.00
Empathy and understanding	52.9 (6.8)	0.84	0.78	0.90	99.96
Pressures	14.5 (9.5)	0.69	0.55	0.81	49.47
Self-acceptance	52.3 (7.1)	0.73	0.62	0.83	73.09
Learning and knowledge	54.2 (6.3)	0.67	0.52	0.81	39.61

**Table 5.** Mean and reliability for domains ‘Emotion and affection’ and ‘Learning and knowledge’ after item removal. Parents with children aged 0–6 months

Domain	Mean (SD)	Estimate (Bayes)	Lower CI (Bayes)	Upper CI (Bayes)	Prob. >0.7
Emotion and affection Item 6 removed	46.5 (3.2)	0.71	0.58	0.83	62.84
Learning and knowledge Item 6 removed	45.3 (5.5)	0.75	0.64	0.86	82.77

## Discussion

We have investigated how the TOPSE for babies works in Norwegian in the context of significant developmental changes occurring in the first year of a child’s life that influence parental experiences and perceptions. By examining TOPSE within this context, we aim to provide insights into its applicability and relevance across various stages of early childhood development.

The Norwegian version of TOPSE for babies proved reliable for most of the six domains. Our analysis showed that there are some differences across the across domains and children’s age groups. The domains of Play and enjoyment and Empathy and understanding demonstrated a good internal consistency for all parents and the parents of children aged 0-6 months. Self-acceptance exhibited a good reliability estimate for all parents and is deemed acceptable for the parents of children aged 0-6 months.

Our findings exhibit similarities and differences when compared to results from other studies. As in our study, the domains of Emotion and Affection (0.60) and Learning and Knowledge (0.67) yielded alpha coefficients which were among the lowest values recorded across all domains examined in the Italian study (Roncaglia et al., 2023). By dropping two items, the reliability of Emotion and affection and Learning and knowledge improved in our study, especially among parents of children aged 0–6 months. Five items in Emotion and affection also demonstrated low variability, which could have implications for the overall reliability of the domain. Conversely, the Bangla study (Ferdowshi, Imran and Trishna, 2021) observed higher alpha values for these two domains (0.81 and 0.91, respectively). Our analysis identified Item 6, ‘Knowing that other people have similar difficulties with their babies makes it easier for me’, from the Learning and knowledge domain as a contributor to the observed low reliability. This was evident across all parents but especially among parents of children aged 0–6 months. While the removal of Item 6, ‘I find it hard to cuddle my baby’ in the Emotion and affection domain yielded a notable improvement in reliability for parents of children aged 0–6 months, the impact on the parents of all age groups was very small. No other item was considered problematic, suggesting that the included variables in this domain might not adequately measure the same underlying construct in our sample.

The Pressures domain showed a higher reliability for all parents, going from an acceptable reliability to questionable for the parents of children aged 0-6 months. The decreased reliability among the youngest children’s parents might indicate potential challenges and stressors unique to the earliest stages of parenthood, that are not sufficiently captured in the questionnaire. While the Pressures domain in the Italian study demonstrated an ‘acceptable’ alpha coefficient (0.67), it was considered ‘good’ in the Bangla study (0.83). Notably, although all domains exhibited ‘acceptable’ or better values in the original validation studies, the three domains we identified as having the lowest reliability in our study consistently showed some of the lowest alpha coefficients in these studies as well (Bloomfield and Kendall 2007; Bloomfield and Kendall 2012; Kendall and Bloomfield 2005).

The items we found to determine the low reliability in our study also significantly contributed to the low reliability observed in the Italian TOPSE for babies. Three items were identified in the item-by-item exclusion in the Italian study to largely determine the low reliability (Roncaglia et al., 2023). These were Item 6 in Emotion and affection ‘I find it hard to cuddle my baby’, Item 3 in Self-acceptance ‘I am not doing that well as a parent’, and Item 6 in Learning and knowledge ‘Knowing that other people have similar difficulties with their babies makes it easier for me’. The Bangla study’s item analysis also identified three items that did not fulfil the acceptable level of corrected item-total correlation (Ferdowshi, Imran, and Trishna, 2021). None of them was the same as in our study. All three items were from the Pressures domain: Item 4, ‘I can say “no” to other people if I don’t agree with them’, Item 5, ‘I can ignore pressure from other people to do things their way’, and Item 6, ‘I do not feel a need to compare myself to other parents’.

In the Italian study, first-time parents experienced a significant improvement in PSE mean scores across the three-time points, while the changes were much smaller for parents with more than one child (Roncaglia et al., 2023). Our study observed no difference in scores for parents with caregiver duties for more than one child versus parents with caregiver duty for only one child. Cultural norms and child-rearing values may also contribute to the variance observed across the TOPSE studies, considering that they were conducted in countries with entirely different cultures.

### Strengths and limitations

A limitation of our study is the small sample due to difficulties experienced in recruiting parents. Therefore, we used a Bayesian framework to accommodate the limited sample size. The small percentage of fathers included in our study is also a limitation, as we could not analyse differences between mothers and fathers. The validation studies of the English TOPSE included very few fathers compared to the Italian study, and Roncaglia et al. (2023) pointed out that this could explain the lower values in the coefficients observed. Having few fathers in the sample is common within studies on PSE. Wittkowski, Dowling and Smith (2016) found that fathers were significantly under-represented in their systematic review of group-based parental interventions’ impact on PSE. Currently, there are numerous PSE measures specifically designed for both mothers and fathers and exclusively for mothers, but there is a notable absence of measures targeting fathers (Wittkowski et al. 2017). Paternal PSE is therefore an area that warrants further investigation.

We did not include educational level in our study, which is pointed out in some of the other studies on TOPSE (Kendall and Bloomfield, 2005; Roncaglia et al., 2023) as a possible factor in the participants’ understanding of the statements. Another limitation of our study is that we did not collect information on where participants accessed the questionnaire. As a result, we are unable to determine the effectiveness of each recruitment site or identify which site was most successful in reaching participants.

Due to the nature of the statements, some of the items may not immediately appear as relevant to parents of infants. This could, for example, apply to the item ‘I am confident my child can come to me if they’re unhappy’. This is also the item that we considered to translate poorly to Norwegian. However, our analysis showed that the difference between the original statement and the statement with a different wording was minimal, although the original was slightly better. Our discussion with the professional translators was critical to arrive at the most appropriate translations. Drawing from the critique of back-translation methodology provided by Ozolins et al. (2020), it becomes evident that the research team’s involvement in the translation process is vital to preserve the intended meaning of the original text and effectively communicates the message in the target language.

In Wittkowski et al.’s (2017) review, the time required for administering TOPSE was noted to exceed the recommended threshold of 10 minutes. However, in our sample, the median response time was 6.6 minutes, indicating that the administration of TOPSE for babies in Norwegian falls within the recommended threshold.

### Impact

TOPSE was developed to evaluate parenting programmes, many of which are delivered within primary healthcare settings. The TOPSE for babies version, tailored for parents of children aged 0–6 months, addresses the specific needs of a group that frequently interacts with primary healthcare services. This underscores the importance of having reliable tools to assess the effectiveness of programmes targeting this group. In addition, we propose that TOPSE has the potential to support public health nurses in primary healthcare by facilitating meaningful conversations with parents. For instance, it could help identify important topics and concerns to address during consultations, thereby enhancing the quality of care provided within the service.

The first 1,000 days, from conception to two years of age, are widely recognized in research, policy, and practice as a critical period for child development (UNICEF, 2020). Early childhood care and education programmes are vital in fostering growth and development during this time. Investing in these programmes enables governments worldwide to uphold their collective responsibility to ensure that every child is supported to reach their full potential (Aguayo and Britto, 2024). In primary care, early intervention and monitoring are essential tools in the role and responsibility of supporting nurturing care for childhood development and enabling children to make the best start in life. Additionally, as recommended by the World Health Organization, caregivers should be supported in providing responsive care, and interventions supporting maternal mental health should be integrated into services for early childhood health and development (WHO, 2020a). Evaluating programmes used in primary care services is in itself also relevant for early childhood development, because it ensures quality in the services and their outcomes (WHO, 2020b). We believe TOPSE can be a valuable tool for healthcare workers and primary care services, aiding them in monitoring, improving, and assuring the quality of their services – aligning with TOPSE’s original purpose. Furthermore, TOPSE could contribute significantly to advancing UNICEF’s goal of ensuring that children worldwide benefit from policies, programmes, and practices that protect, promote, and support child development (UNICEF, 2023). This critical focus is promoted in primary care services’ guidelines and national investments in Norway and globally.

## Conclusion

The Norwegian version of TOPSE for babies proved to be a reliable tool. Removing one item in each of the domains showing lower alpha coefficients led to improved reliability. Consequently, our findings suggest that minor changes make the Norwegian version a reliable tool for assessing PSE, and the tool appears to perform adequately for parents of children aged 0–6 months, for which this version of TOPSE was specifically made. While our findings are promising, further research is warranted to establish a more robust evaluation of the tool across both mothers and fathers, parental age and socioeconomic groups, to name a few.

## References

[ref1] Aguayo VM and Britto PR (2024) The first and next 1000 days: a continuum for child development in early life. The Lancet 404(10467), 2028–2030. 10.1016/S0140-6736(24)02439-5 39571588

[ref2] Ardelt M and Eccles JS (2001) Effects of mothers’ parental efficacy beliefs and promotive parenting strategies on inner-city youth. Journal of Family Issues 22(8), 944–972.

[ref3] Bloomfield L and Kendall S (2007) Testing a parenting programme evaluation tool as a pre- and post-course measure of parenting self-efficacy. Journal of Advanced Nursing 60(5), 487–493. 17973712 10.1111/j.1365-2648.2007.04420.x

[ref4] Bloomfield L and Kendall S (2012) Parenting self-efficacy, parenting stress and child behaviour before and after a parenting programme. Primary Health Care Research & Development 13(4), 364–372. 22464178 10.1017/S1463423612000060

[ref5] Bot SD , Terwee CB , van der Windt DA , Bouter LM , Dekker J and de Vet H C (2004) Clinimetric evaluation of shoulder disability questionnaires: a systematic review of the literature. Annals of the Rheumatic Diseases 63(4), 335–341. 15020324 10.1136/ard.2003.007724 PMC1754942

[ref7] Fang Y , Boelens M , Windhorst DA , Raat H and van Grieken A (2021) Factors associated with parenting self-efficacy: A systematic review. Journal of Advanced Nursing 77(6), 2641–2661. 33590585 10.1111/jan.14767 PMC8248335

[ref8] Ferdowshi N , Imran MA and Trishna TA (2021) Adaptation of the tool to measure parenting self-efficacy (topse) in Bangladesh. Dhaka University Journal of Biological Sciences 30(2), 169–177.

[ref9] George D and Mallery P (2003) SPSS for Windows step by step: A simple guide and reference. 11.0 Update (4th ed.). Boston, MA: Allyn & Bacon.

[ref10] Gohel D & Skintzos P (2023). *flextable: Functions for Tabular Reporting*. R package version 0.6.10. Retrieved from https://CRAN.R-project.org/package=flextable

[ref15] Jones TL and Prinz RJ (2005) Potential roles of parental self-efficacy in parent and child adjustment: a review. Clinical Psychology Review 25 (3), 341–363. 15792853 10.1016/j.cpr.2004.12.004

[ref11] Kendall S and Bloomfield L (2005) Developing and validating a tool to measure parenting self-efficacy. Journal of Advanced Nursing 51(2), 174–181. 15963189 10.1111/j.1365-2648.2005.03479.x

[ref12] Kendall S (2023) TOPSE Development. Available: https://www.topse.org.uk/site/topse-development/ (Accessed 28.10.2023).

[ref14] Nystrom K and Ohrling K (2004) Parenthood experiences during the child’s first year: literature review. Journal of Advanced Nursing 46(3), 319–330. 15066113 10.1111/j.1365-2648.2004.02991.x

[ref16] Ozolins U , Hale S , Cheng X , Hyatt A , Schofield P (2020) Translation and back-translation methodology in health research – a critique. Expert Review of pharmacoeconomics & Outcomes Research 20(1), 69–77. 32089017 10.1080/14737167.2020.1734453

[ref32] Øvrelid E , Bygstad B and Thomassen G (2021) TSD: a research platform for sensitive data. Procedia Computer Science 181, 127–134.

[ref17] Pfadt JM , van den Bergh D , Goosen J (2023) *_Bayesrel: Bayesian Reliability Estimation_. R package version 0.7.7*. Retrieved from https://CRAN.R-project.org/package=Bayesrel

[ref18] Pfadt JM , van den Bergh D , Sijtsma K & Wagenmakers E (2023) A tutorial on Bayesian single-test reliability analysis with JASP. Behavior Research Methods 55(3), 1069–1078. 35581436 10.3758/s13428-021-01778-0 PMC10126026

[ref19] R Core Team. (2022) R: A language and environment for statistical computing. R Foundation for Statistical Computing, Vienna, Austria. URL: https://www.R-project.org/

[ref20] Roncaglia F , Bonvicini L , Kendall S , Panza C , Ferraroni M and Giorgi Rossi P (2023) Validation of the Italian version of the tool to measure parenting self-efficacy (TOPSE) questionnaire using data from an intervention study. Child: Care, Health and Development 49(1), 189–200. 35817590 10.1111/cch.13032

[ref21] Sjoberg DD , Whiting K , Curry M , Lavery JA and Larmarange J (2021). Reproducible summary tables with the gtsummary package. The R Journal 13(1), 570–580.

[ref22] Sokolovic N , Grujic S and Pajic S (2022). Evaluation of the support, not perfection parenting program in Serbia. European Journal of Developmental Psychology 19(3), 365–382.

[ref23] Terwee CB , Bot SD de Boer MR , van der Windt DA , Knol DL , Dekker J , Bouter LM and de Vet HCW (2007) Quality criteria were proposed for measurement properties of health status questionnaires. Journal of Clinical Epidemiology 60(1), 34–42. 17161752 10.1016/j.jclinepi.2006.03.012

[ref24] United Nations Children’s Fund. (2020) Nutrition for every child. UNICEF nutrition strategy 2020-2030. UNICEF. https://www.unicef.org/media/106511/file/%20Nutrition%20Strategy%202020-2030%20(Document)%20-%202021%20Edition.pdf

[ref25] United Nations Children’s Fund. (2023) Early Childhood Development – UNICEF vision for every child. UNICEF. https://www.unicef.org/media/145336/file/Early%20Childhood%20Development%20-%20UNICEF%20Vision%20for%20Every%20Child.pdf

[ref26] University of Oslo. (2024) Services for sensitive data (TSD). Available at: https://www.uio.no/english/services/it/research/sensitive-data/index.html (Accessed: 03.05.24).

[ref27] World Health Organization. (2010) *WHODAS 2.0 Translation package (Version 1.0). Translation and linguistic evaluation protocol and supporting material* [PDF]. Available at: https://terrance.who.int/mediacentre/data/WHODAS/Guidelines/WHODAS%202.0%20Translation%20guidelines.pdf (Accessed February 23, 2024).

[ref28] World Health Organization. (2020a) Improving early childhood development. WHO. https://iris.who.int/bitstream/handle/10665/331306/9789240002098-eng.pdf?sequence=1 32200595

[ref29] World Health Organization. (2020b) Monitoring children’s development in primary care services. WHO. https://iris.who.int/bitstream/handle/10665/335832/9789240012479-eng.pdf?sequence=1

[ref30] Wittkowski A , Dowling H and Smith DM (2016) Does engaging in a group-based intervention increase parental self-efficacy in parents of preschool children? A systematic review of the current literature. Journal of Child and Family Studies 25(11), 3173–3191. 27795657 10.1007/s10826-016-0464-z PMC5061830

[ref31] Wittkowski A , Garrett C , Calam R and Weisberg D (2017) Self-report measures of parental self-efficacy: a systematic review of the current literature. Journal of Child and Family Studies 26(11), 2960–2978. 29081640 10.1007/s10826-017-0830-5 PMC5646137

